# Honeybee Colony Disorder in Crop Areas: The Role of Pesticides and Viruses

**DOI:** 10.1371/journal.pone.0103073

**Published:** 2014-07-21

**Authors:** Noa Simon-Delso, Gilles San Martin, Etienne Bruneau, Laure-Anne Minsart, Coralie Mouret, Louis Hautier

**Affiliations:** 1 Beekeeping Research and Information Centre, Louvain la Neuve, Belgium; 2 Environmental Sciences, Copernicus Institute, Utrecht University, Utrecht, The Netherlands; 3 Plant Protection and Ecotoxicology Unit, Life Sciences Department, Walloon Agricultural Research Centre, Gembloux, Belgium; Universidade de São Paulo, Faculdade de Filosofia Ciências e Letras de Ribeirão Preto, Brazil

## Abstract

As in many other locations in the world, honeybee colony losses and disorders have increased in Belgium. Some of the symptoms observed rest unspecific and their causes remain unknown. The present study aims to determine the role of both pesticide exposure and virus load on the appraisal of unexplained honeybee colony disorders in field conditions. From July 2011 to May 2012, 330 colonies were monitored. Honeybees, wax, beebread and honey samples were collected. Morbidity and mortality information provided by beekeepers, colony clinical visits and availability of analytical matrix were used to form 2 groups: healthy colonies and colonies with disorders (n = 29, n = 25, respectively). Disorders included: (1) dead colonies or colonies in which part of the colony appeared dead, or had disappeared; (2) weak colonies; (3) queen loss; (4) problems linked to brood and not related to any known disease. Five common viruses and 99 pesticides (41 fungicides, 39 insecticides and synergist, 14 herbicides, 5 acaricides and metabolites) were quantified in the samples.The main symptoms observed in the group with disorders are linked to brood and queens. The viruses most frequently found are Black Queen Cell Virus, Sac Brood Virus, Deformed Wing Virus. No significant difference in virus load was observed between the two groups. Three acaricides, 5 insecticides and 13 fungicides were detected in the analysed samples. A significant correlation was found between the presence of fungicide residues and honeybee colony disorders. A significant positive link could also be established between the observation of disorder and the abundance of crop surface around the beehive. According to our results, the role of fungicides as a potential stressor for honeybee colonies should be further studied, either by their direct and/or indirect impacts on bees and bee colonies.

## Introduction

The evolution of pollinator populations has been the subject of an increasing number of studies, most of them showing worrying negative trends [1]–[4]. Furthermore, beekeepers have long been notifying enhanced bee winter losses and disorders [5]–[8]. Bees contribute to ecosystem services and their decline thus threatens pollination of both wild and cultured plants, endangering biodiversity, food and fibre production [9], [10]. This decline may also have an impact on the production of other goods with pharmacological uses (e.g. honey, propolis) [11], [12], and scientific and technological inspiration (e.g. development of visual guided flight robotics) [13]. Bees are also part of our culture (e.g. culinary, hobby occupation, etc.), contributing to the dynamism of rural and urban areas [14] and providing a source of inspiration and well-being for many [15].

Belgium shares the trends observed worldwide both in terms of wild and reared pollinators [8], [16], with enhanced winter mortality observed from 1999 [8], [17], [18]. However, apart from colony mortality, Walloon beekeepers have reported a number of imprecise symptoms: colony weakness, mainly in spring; fast renewal of young queens; rapid loss of individuals in the colony, mainly foragers, or slow loss of individuals in the colony. In many cases brood and food remains in the colony. Sometime a small cluster of bees with the queen survives [17]. Some beekeepers described unspecific brood abnormalities not characteristic to any known disease (Baudoin and Lequeux, pers. com.). Other studies describe similar mortality trends [8], [19], [20] as well as unspecific symptoms: increased colony mortality and/or weakening [21]–[23], queen failure [21], [23]–[25] or honey yield reduction [21].

Multifarious factors have been proposed to provide a cause to such a phenomenon. Climate change is proposed as one of them, together with a decrease of genetic variability of the bee colonies, electromagnetic radiation, pathogens and parasites or the impact of intensive agricultural systems (nutritional lack, GM plants or pesticides) [26]. Previous studies developed in the region discard some of these factors as causes of bee losses, specifically *Nosema* spp and American Foulbrood [27]. However, pesticide residues and certain viruses were detected in bee colonies [28], [27]. These elements were the most relevant to us.

Countless studies have shown lethal and sub-lethal effects of pesticides on bees [29]–[31]. Insecticides are often the most studied for obvious reasons. However other substances (fungicides, herbicides) deserve analysis for their specific toxicity, individually or in synergy with other substances [32], [33] or pathogens [34]–[36], or their extensive exposure given their large scale and/or repeated use. Indeed, residue analyses show that these types of substance are found in the hives, even though the crops in which they were applied would suggest no bee exposure [37]–[40].

Beside pesticides, bee viruses are often mentioned as a putative cause of decline of the colonies, or at least to reside among the presumed multi-factorial causes [41]. In Europe, at least 12 viruses infecting bees have been compiled [21]. Many honeybee viruses commonly occur in seemingly healthy populations that continue to run well. Viruses may remain latent and confined within certain cells or tissues with no active replication and no disruption of cellular function. Likewise, they may be replicating at low level in permissive cells but in non-vital sites or in honeybee life stages that do not exhibit any symptoms or obvious pathology [21], [42], [43]. Nevertheless, two viruses, ABPV and DWV, are able of inducing serious disorders to honeybees and have been shown to cause -in association with *V. destructor* - winter losses in Germany [23].

In this paper, we first studied the relationship of in-hive viral prevalence as well as the presence of pesticide residues on honeybee colonies’ health. As a next step, we focused on the relationship between the environment surrounding the monitored apiaries and the health condition of their colonies.

## Materials and Methods

### Field work – colony follow up

A group of voluntary beekeepers were requested to participate in the study. All of them shared the following criteria: (1) have a minimum of 5 production colonies per apiary in June 2011; (2) regularly follow up the health status and development of their colonies; and (3) monitor the varroa infestation level and carry out officially recommended varroa treatments (treatment in July-August with veterinary medicaments based on thymol and winter treatment with veterinary medicaments based on oxalic acid). A total of 330 colonies distributed among 66 apiaries (5 colonies/apiary) in Walloon and Brussels regions (Belgium) were followed. In those apiaries composed of 5 or more colonies, the 5 well-developed colonies at the beginning of the study were selected.

Colonies received three visits along the study, the first one from mid-July to mid-August 2011, the second one from mid-September to mid-October 2011 and the third one from March to April 2012. State official beekeeping technicians specialised in bee health were trained in the framework of the project in order to minimise as far as possible the variability of the results due to handling and observations. For each of the visits, beekeepers were requested to fulfil a form asking information about the health history of the apiary and of the followed-up colonies, as well as about their beekeeping practices. Honeybee colony disorder symptoms were reported in the questionnaire. These include the following symptoms for which no causal agent could obviously be identified:

1. dead and disappeared colonies : (1a) death of part or the whole colony, where dead bees can be found close to the hive or inside it. Beekeepers also describe the (1b) disappearance of part or the whole colony, leaving behind food reserves and brood, a phenomenon similar to the one described in North-American apiaries (Colony Collapse Disorder (CCD) [24];
2. weak colonies : weakening of the colony, showing in occasions a slow development in spring under optimal conditions (e.g. optimal weather, low varroa pressure, etc) with as consequence the loss of the spring production, but in occasions showing abnormally small colonies or colonies with low activity;
3. queen loss : replacement of young queens sometimes leading to queen-less colonies or interruption of the egg-laying activity of the queen [17];
4. problems linked to brood and not related to any known disease.

The form also included requests about other symptoms typically linked to known diseases (e.g. diarrhoea, mummified larvae, varroa presence, presence of deformed wing bees, etc). Each of the questions requested further characterisation of the symptoms (e.g. population size, population dynamics, bees behaviour, etc.).

In addition, a thorough clinical inspection was carried out for each colony in the mentioned period. Special attention was given to the strength of the colony in terms of bee numbers, brood surface and reserves content, the presence of the queen and the varroa infestation level. Finally, two different samples were collected before and after the winter: (1) in-hive bees (minimum of 100 bees); (2) a section of the frame containing beebread and honey (about 100 cm^2^). Samples were collected in hermetic plastic bags, cooled after collection and stored at −20°C.

### Case choice – hierarchical sample clustering

Information available from bee colonies comprised field observations, beekeeper answers to the questionnaire and results from the analyses of the samples of beekeeping matrices (honey, beebread, wax and bees). Colonies for which this information was available were considered for our analyses. Colonies with well identified problems (heavy varroa infestation, lack of food or drone-laying queens) were discarded. After that, two groups were made: a group with disorders and another with healthy colonies. In order to constitute these groups and to limit variations due to potential different bee management, a hierarchical classification was made. The criteria used were the amount of food stores before the winter, the year of colony creation, the subspecies and the age of the queen. This classification was made by using Ward aggregation method. This method allows to build group with the lower variation within the group and the higher variation between groups [44].

### Virus analyses

The viruses under investigation were the Black Queen Cell Virus (BQCV), the Chronic Bee Paralysis Virus (CBPV), the Acute Bee Paralysis Virus (ABPV), the Deformed Wings Virus (DWV), and the Sac Brood Virus (SBV). Viral analyses were conducted on the honeybee workers collected before the winter, during the first and second periods (July–August and September–October). The samples were analysed with a quantitative RT-PCR by the National Bee Unit laboratory, Food and Environment Research Agency (Sand Hutton, York, United-Kingdom).

Total Nucleic acid (TNA) was extracted from 60 bees homogenised for 12 minutes with 20 ml GITC Lysis Buffer (5 M Guandine Thiocynate, 0.05 M Tris base, 0.02 M EDTA, pH 8.0) in a 30 ml bottle containing 3, 7/16″ ball bearings. GITC Lysis buffer also contained 17.3 mM SDS buffer (173 mM Sodium dodecyl sulphate (SDS) in 100 ml MGW). The SDS buffer is added prior to use in warmed GITC Lysis Buffer. The homogenate was then incubated at 65°C for 40 minutes and then spun at 6189 g for 5 minutes. Polypropylene 96 -deep well plates (DT850301 Elkay Laboratory Products Ltd) were prepared as follows (1 well/extract); plate A: 800 µl extract, and 100 µl MagneSil™ beads (MD1441, Promega); plate B: 1 ml of GITC wash buffer (5 M Guandine Thiocynate, 0.05 M Tris base); plates C, D, E: 1 ml 70%v/v ethanol (E/00665DF/17, Fisher Scientific); plate F: 1 ml 5 M Betaine (B2629, Sigma), plate G, 300 ul 1×TE Buffer (stock 100X TE (EC-862 National Diagnostics). Plates were loaded onto the Kingfisher Flex and processed as follows: Plate A – Bind 5 mins (fast dual mix), Plate B – Wash 3 mins (fast dual mix), Plate C, D, E – Wash 2 mins (fast dual mix), collect beads at 1 min intervals, Plate F – Wash 20 secs (medium), without releasing beads, Plate G – Mix 1 min then incubate at 65°C for 5 mins with mixing. All steps of the process are looped through twice. TNA was collected from plate G of each reaction and stored at −80°C prior to use in real-time PCR.

Reactions were set up in 96 well reaction plates using Absolute Blue QPCR ROX mix (AB-4139, Thermo Scientific) following the protocols supplied. 0.1 mM of 0.1 M Dithiothreitol (165680250, Arcos Organics) and 0.33 unit of MMLV (EPO441: Fermentas) were added to each reaction. The primers (Table 1) were all used at 400 nM and probes at 200 nM final concentration. Total nucleic acid (5 µl) was added to each reaction, giving a final reaction volume of 20 µl. Plates were cycled for 48°C/30 min, 95°C/10 min and 40 cycles of 60°C/1 min, 95°C/15 sec within the 7900 HT Sequence Detection System (Applied Biosystems), using real time data collection. The results were recorded as the cycle threshold (Ct) or cycle number after which a significant accumulation of florescence over the baseline was observed; an average (of duplicate wells) Ct value below 40 was regarded as a positive result with a threshold ΔRn setting of 0.2. Given the absence of internal standard, we assumed that the extraction method had the same efficiency towards bee DNA/RNA and viral RNA.

**Table 1:** List of primers used for virus analyses.

Target	Primer Name	Sequence (5′–3′)
BQCV	BQCV 9195F	GGT GCG GGA GAT GAT ATG GA
	BQCV 8265R	GCC GTC TGA GAT GCA TGA ATA C
	BQCV 8217T	TTT CCA TCT TTA TCG GTA CGC CGC C
SBV	SBV 311F	AAG TTG GAG GCG CGy AAT TG
	SBV 380R	CAA ATG TCT TCT TAC dAG AGG yAA GGA TTG
	SBV 331T (MGB)	CGG AGT GGA AAG AT
CPV	CPV 304F	TCT GGC TCT GTC TTC GCA AA
	CPV 371R	GAT ACC GTC GTC ACC CTC ATG
	CPV 325T	TGC CCA CCA ATA GTT GGC AGT CTG C

Virus primers and probes used for all pathogens tested have been previously described in Chantawannakul et al., 2006 [45]. Additional APBV primers are described in Martin et al., 2012. [46].

### Pesticide analyses

Samples collected before the winter were sent to Eurofins Chemiphar NV, Brugge, Belgium and analysed by SOFIA GmbH Chemisches Labor für Softwareentwicklung und Intelligente Analytik, Berlin, Germany. Two multi-residues methods (SF146 and SF150) were used searching for 99, 93 and 96 pesticides residues in wax (54 samples), beebread (108 samples) and honey (107 samples – one sample did not contain enough matrix) respectively (LOQ in Table 2).

**Table 2:** Active ingredients or metabolite included in the multi-residue analyses by beekeeping matrix (A = Acaricide; F = Fungicide; H = Herbicide; I = Insecticide; S = Synergist).

Active ingredient or metabolite*	Class	LOQ (mg/kg)		
		Wax	Beebread	Honey
2,4-D	H	0.1	–	0.01
Abamectin	I	0.1	0.1	0.005
Acetamiprid	I	0.1	0.1	0.005
Aldicarb	I	0.1	0.1	0.005
Alpha-cypermethrin	I	0.1	0.1	0.005
Amitraz	A	0.1	0.1	0.01
Azoxystrobin	F	0.1	0.1	0.005
Bentazone	H	0.2	–	0.02
Benthiavalicarb	F	0.1	0.1	0.005
Beta-cyfluthrin	I	0.1	0.1	0.01
Bifenthrin	I	0.1	0.1	0.005
Boscalid	F	0.1	0.1	0.005
Captan	F	0.1	0.1	0.01
Carbaryl	I	0.1	0.1	0.005
Chlorpyriphos	I	0.1	0.1	0.005
Chlorpyriphos-methyl	I	0.1	0.1	0.005
Chlorothalonil	F	0.1	0.1	0.005
Clothianidin	I	0.1	0.1	0.01
Coumaphos	A	0.1	0.1	0.05
Coumaphos oxon *	A	0.1	0.1	–
Coumaphos phenolic*	A	0.1	0.1	–
Cyazofamid	F	0.2	0.2	0.02
Cyfluthrin	I	0.1	0.1	0.01
Cymoxanil	F	0.1	0.1	0.01
Cypermethrin	I	0.1	0.1	0.005
Cyproconazole	F	0.1	0.1	0.005
Cyprodinil	F	0.1	0.1	0.01
DDT	I	0.1	0.1	0.005
Deltamethrine	I	0.1	0.1	0.005
Dichlorprop-P	H	0.1	–	0.01
Difenoconazole	F	0.1	0.1	0.005
Diflubenzuron	I	0.2	0.2	0.02
Dimethenamid-P	H	0.1	0.1	0.005
Dimethoate	I	0.1	0.1	0.005
Dimethomorph	F	0.1	0.1	0.01
Dimoxystrobin	F	0.1	0.1	0.005
Epoxiconazole	F	0.1	0.1	0.005
Esfenvalerate	I	0.1	0.1	0.005
Ethofumesate	H	0.5	0.5	0.05
Famoxadone	F	0.1	0.1	0.01
Fenhexamid	F	0.1	0.1	0.005
Fenoxycarb	F	0.1	0.1	0.005
Fenpropidin	F	0.1	0.1	0.01
Fenpropimorph	F	0.1	0.1	0.005
Fipronil	I	0.1	0.1	0.01
Flonicamid	I	2	2	0.2
Fluazinam	F	0.1	0.1	0.01
Flufenacet	H	0.1	0.1	0.01
Fluopicolide	F	0.1	0.1	0.01
Flusilazole	F	0.1	0.1	0.005
Heptenophos	I	0.1	0.1	0.01
Imidacloprid	I	0.1	0.1	0.005
Indoxacarb	I	0.1	0.1	0.005
Iprodione	F	0.1	0.1	0.005
Kresoxim-methyl	I	0.1	0.1	0.005
Lambda-cyhalothrin	I	0.1	0.1	0.005
Lindane	I	0.1	0.1	0.005
Linuron	H	0.1	0.1	0.005
MCPA	H	0.1	–	0.01
Mecoprop-P	H	0.1	–	0.01
Metalaxyl-M	F	0.1	0.1	0.005
Metamitron	H	0.1	0.1	0.005
Metconazole	F	0.2	0.2	0.02
Methiocarb	I	0.1	0.1	0.005
Methoxyfenozide	F	0.1	0.1	0.005
Metribuzin	H	0.1	0.1	0.005
Penconazole	F	0.1	0.1	0.005
Pendimethalin	H	0.1	0.1	0.005
Permethrin	I	0.2	0.2	0.02
Picoxystrobin	F	0.1	0.1	0.005
Piperonyl butoxide	S	0.1	0.1	0.01
Pirimicarb	I	0.1	0.1	0.005
Prochloraz	F	0.1	0.1	0.005
Propamocarb	F	0.1	0.1	0.005
Propiconazole	F	0.1	0.1	0.01
Propyzamide	F	0.1	0.1	0.01
Pymetrozine	I	0.1	0.1	0.005
Pyraclostrobin	F	0.1	0.1	0.005
Pyrethrin	I	0.2	0.2	0.02
Pyrimethanil	F	0.1	0.1	0.005
Rotenone	I	0.1	0.1	0.01
Spinosad	I	0.1	0.1	–
Spirodiclofen	I	0.1	0.1	0.01
Spirotetramat	F	0.1	0.1	0.01
Spiroxamine	F	0.1	0.1	0.005
Sulcotrione	H	0.1	–	0.01
Tau-fluvalinate	IA	0.1	0.1	0.01
Tebuconazole	F	0.1	0.1	0.005
Tebufenozide	I	0.1	0.1	0.005
Tefluthrine	I	0.1	0.1	0.005
Terbuthylazine	H	0.1	0.1	0.01
Tetraconazole	F	0.1	0.1	0.01
Thiabendazole	F	0.1	0.1	0.005
Thiacloprid	I	0.1	0.1	0.005
Thiamethoxam	I	0.1	0.1	0.005
Thiophanate-methyl	F	0.1	0.1	0.005
Trifloxystrobin	F	0.1	0.1	0.01
Zeta-cypermethrin	I	0.1	0.1	0.005
Zoxamide	F	0.1	0.1	0.01

For SF146 method, 10 ml of water was added to 10 g of samples. Methanol was added and the preparation was mixed. The mixture was filtered and centrifuged. Next, for analysis by gas chromatography coupled with mass spectrometry (GC-MS), the filtrate was mixed with sodium chloride and ethyl acetate solution 1∶1 (v/v). All was dried with sodium sulfate and filtrated. This filtrate was then concentrated and analysed by GC-MS. For analysis with liquid chromatography coupled with tandem mass spectrometry (LC-MS/MS), 5 ml of sample were transferred in ChemElut column and eluted with dichloromethane. After concentration, 1 ml of water-methanol 1∶1 (v/v) was added and this solution was analysed by LC-MS/MS. For the wax, some modifications were made. For GC-MS, sodium chloride was replaced by hexane to dissolve wax and the mixture was frozen until a precipitate appears. For LC-MS/MS, 0.2 g of sample were extracted with 30 ml of mixture of naphtha:acetonitrile 1∶2 (v/v). 10 ml of acetonitrile phase was isolated and concentrated before the analysis by LC-MS/MS.

For SF150 method, 10 ml of water was added to 10 g of samples as well as methanol and hydrochloric acid. All was mixed, filtrated and centrifuged. A solution of sodium chloride (20%) was added and 5 ml of sample were transferred in ChemElut column and eluted with dichloromethane. After concentration, 1 ml of water-methanol 1∶1 (v/v) was added and this solution was analysed by LC-MS/MS.

### Descriptive analyses

A descriptive analysis of virus prevalence and pesticide residues was first carried out. The relationship between disorder and these stressing factors was then assessed. For each virus type, a linear model (two way ANOVA) was used, with the cycle threshold value as dependent variable and the visit (first or second), group (with disorder or healthy) and their interaction as explanatory variables. For the pesticides residues, we used a similar model with the total number of residues as dependent variable and type (fungicide or insecticide/acaricide), group (with disorder or healthy) and their interaction as explanatory variable. Based on these models, we constructed a contrast matrix to test explicit post-hoc hypotheses (e.g. compare virus load between groups within each visit…). As several dependent variables had a strongly asymmetric distribution (i.e. non-normal), we computed all p-values by permutation (n = 1000). We applied a Bonferoni correction on the post-hoc tests p-values to take into account the multiplicity of the tests.

### Relationship between bee colony disorders and potential stressors or surrounding environment of the apiary

We used two separate generalized linear mixed models with a binomial distribution and logit link function. In both models, the “group” (with disorder or healthy) was used as dependent binary variable and the apiary was used as random effect (grouping factor) to take into account the non-independence between colonies from the same apiary and allow the intercept to vary between apiaries [47].

With the first model, we explored the relationship between the probability of disorder in a colony and potential stress factors, i.e. pesticides or viruses total load into the colonies. We used as explanatory variables the total number of fungicides residues, the total number of insecticides or acaricides (pooled together) residues, the total number of viruses detected for both visits and all first level interactions between these three explanatory variables. The insecticides and acaricides were pooled together because the most frequently found active ingredient, namely tau-fluvalinate, is authorized in Belgium for both purposes.

With the second model, we investigated the relationship between the probability of disorder in a colony and the structure of the agricultural landscape around the beehive. The surfaces of all different kinds of agricultural soil occupancy in a circle with a radius of 1500 m around each apiary were calculated according to the information provided by the farmers to the Walloon administration (Land Parcel Identification System). The different soil occupancy categories were pooled into two categories defined according to the potential frequency of plant protection product use: (1) grasslands (low pesticides use), (2) crops *sensu lato* (potentially higher pesticides use), including major crops (mainly: cereals, potatoes, beet, oilseed rape, maize, flax, …) but also surfaces dedicated to fruit or vegetables production, fodder production (mainly legumes) and to horticulture. The surfaces of these two land use options, grasslands and crops (*sensu lato*), were used as explanatory variables in the model. The 1500 m radius was chosen accordingly to the mean pollen and nectar collecting distance for the honeybee [48], [49]. As the agricultural soil occupancy data was available only for Wallonia, we eliminated from the analysis the apiaries whose buffer had less than 70% of their surface within the administrative boundaries of Wallonia (n = 2).

We used Likelihood Ratio (LR) Tests to evaluate the significance of the explanatory variables [47], [50]. We respected the marginality rules, i.e. all main effects were tested after removing from the model the interactions in which they are involved [51].

All analyses were performed with R (R Core Team 2013) and the mixed models were fitted with the package lme4 [52].

## Results

### Description of honeybee colony disorders

**Figure 1:**
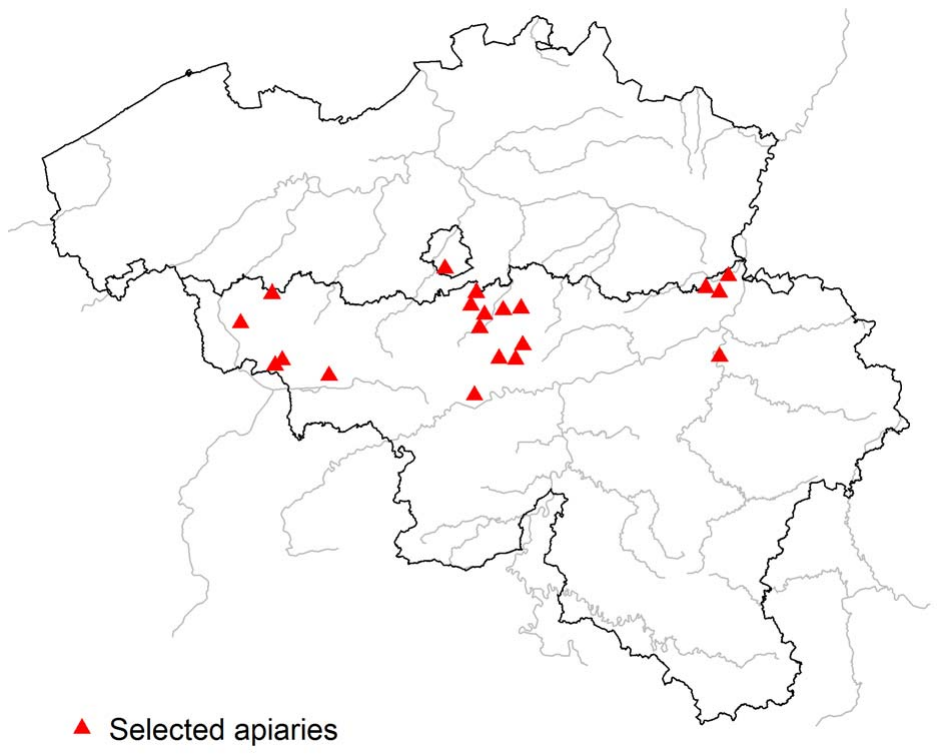
Spatial distribution of selected apiaries.

We gathered all sources of information, i.e. questionnaires, analytical matrices and information from clinical visits, for 173 colonies. After the clustering, 54 colonies coming from 21 apiaries (Figure 1) were considered for the study: 25 presenting bee disorders and 29 not presenting them, defined as healthy group. Their data and samples were collected by 8 beekeeping technicians.

In the group presenting disorders, 6 colonies were dead or unviable -with only a handful of bees remaining with the queen- at the spring visit (Table 3). Pre-wintering weakness, winter worker losses, late-season re-queening were reported in these cases. One colony was dead due to queen failure without re-queening in spring. Furthermore, a number of colonies were weak in spring. They were characterised by a low number of individuals and a slow development. In total, nine colonies lost their queens during the study, five of them leading to queenless colonies. These were considered as “disorder colonies” in the framework of the study. Finally, brood abnormalities not linked to known disease were observed in 10 colonies, most of them both before and after the winter.

**Table 3:** Symptoms observed in the group with disorders.

Symptoms of disorder	Frequency
Mortality	2*
Weakening	3
Queen failure	5
Brood problems	9
Mortality+Weakening	2
Mortality+Weakening+Queen failure	2
Mortality+Queen failure	1
Queen failure+Brood problems	1
Total	25

* One of these colonies not considered in the model due to a lack of viral results.

Symptoms of other diseases or bee parasite observation remained low within the selected colonies. Wax moths were observed in two cases, one in each group. *Varroa destructor* was present in all colonies of the study. All of them were treated according to official veterinary advice. Diarrhoea was observed in one of the colonies showing brood abnormalities. Anatomical modifications (i.e. deformed or undeveloped wings, small abdomens) were observed in 6 colonies, half of them being healthy and half of them showing disorders. No symptoms of any of the foulbroods were observed.

### Virus content

**Figure 2:**
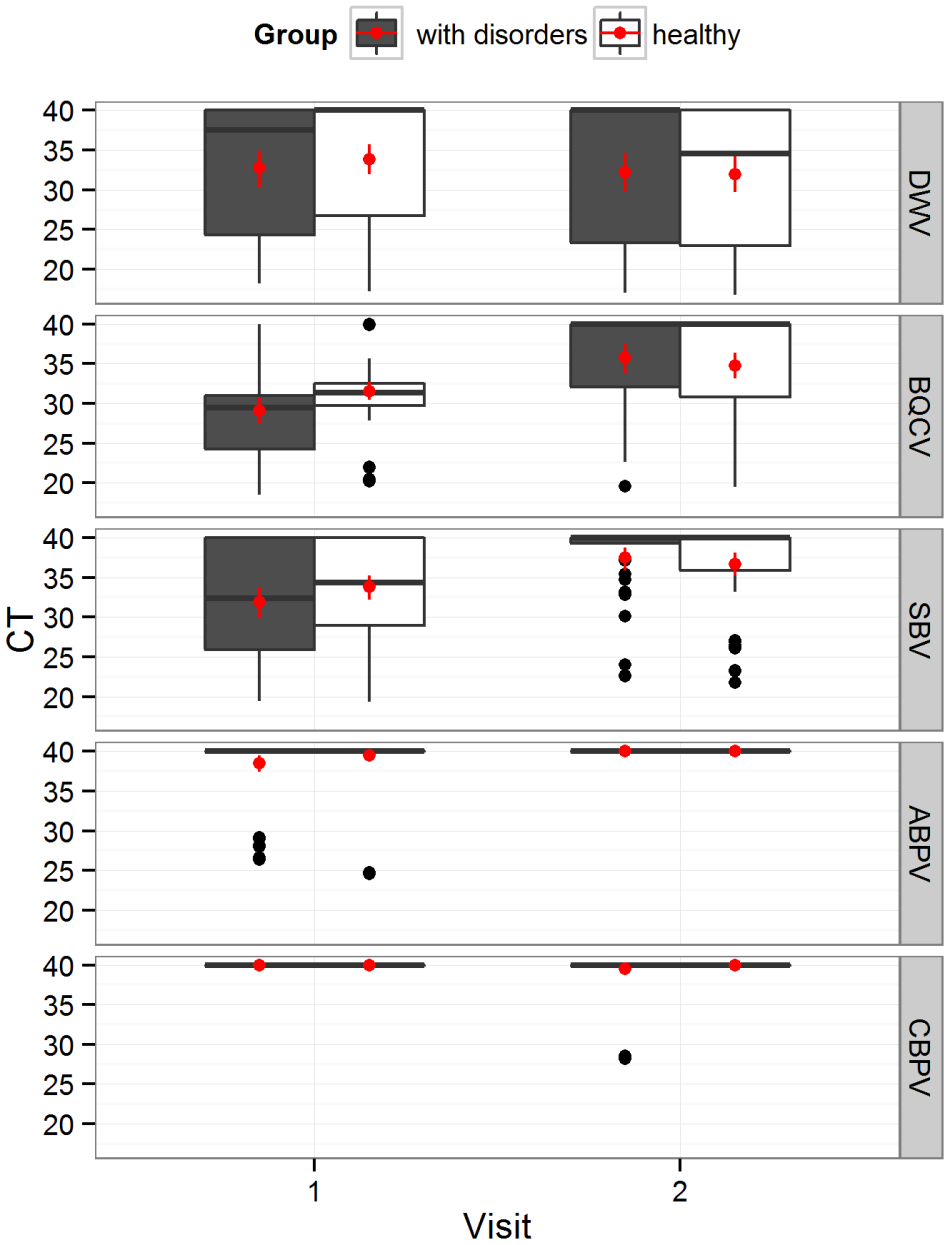
Virus content according to the Cycle Threshold (CT) for the groups “with disorder” and “healthy”. Boxplot of Cycle Threshold for the first and second visits (visit 1 - mid-July to mid-august and visit 2 - mid-September to mid-October) and the group with disorders (grey, n = 24 colonies) and the healthy one (white, n = 29 colonies). Deformed Wings Virus (DWV), Black Queen Cell Virus (BQCV), SacBrood Virus (SBV), Acute Bee Paralysis Virus (ABPV), Chronic Bee Paralysis Virus (CBPV). In red, mean with confidence interval estimated by boostrap method. Note: CT values below 40 were regarded as positive results and the lowest CT values correspond to the higher virus contents.

One bees sample from the “disorder” group could not be analysed due to a lack of sufficient matrix. The three most abundantly found viruses were the Deformed Wings Virus (DWV), the Black Queen Cell Virus (BQCV) and the Sac Brood Virus (SBV) (Figure 2). The Acute Bee Paralysis Virus (ABPV) and the Chronic Bee Paralysis Virus (CBPV) were found only in a very limited number of samples and did not allowed particular statistical analyses on these two viruses.

**Figure 3:**
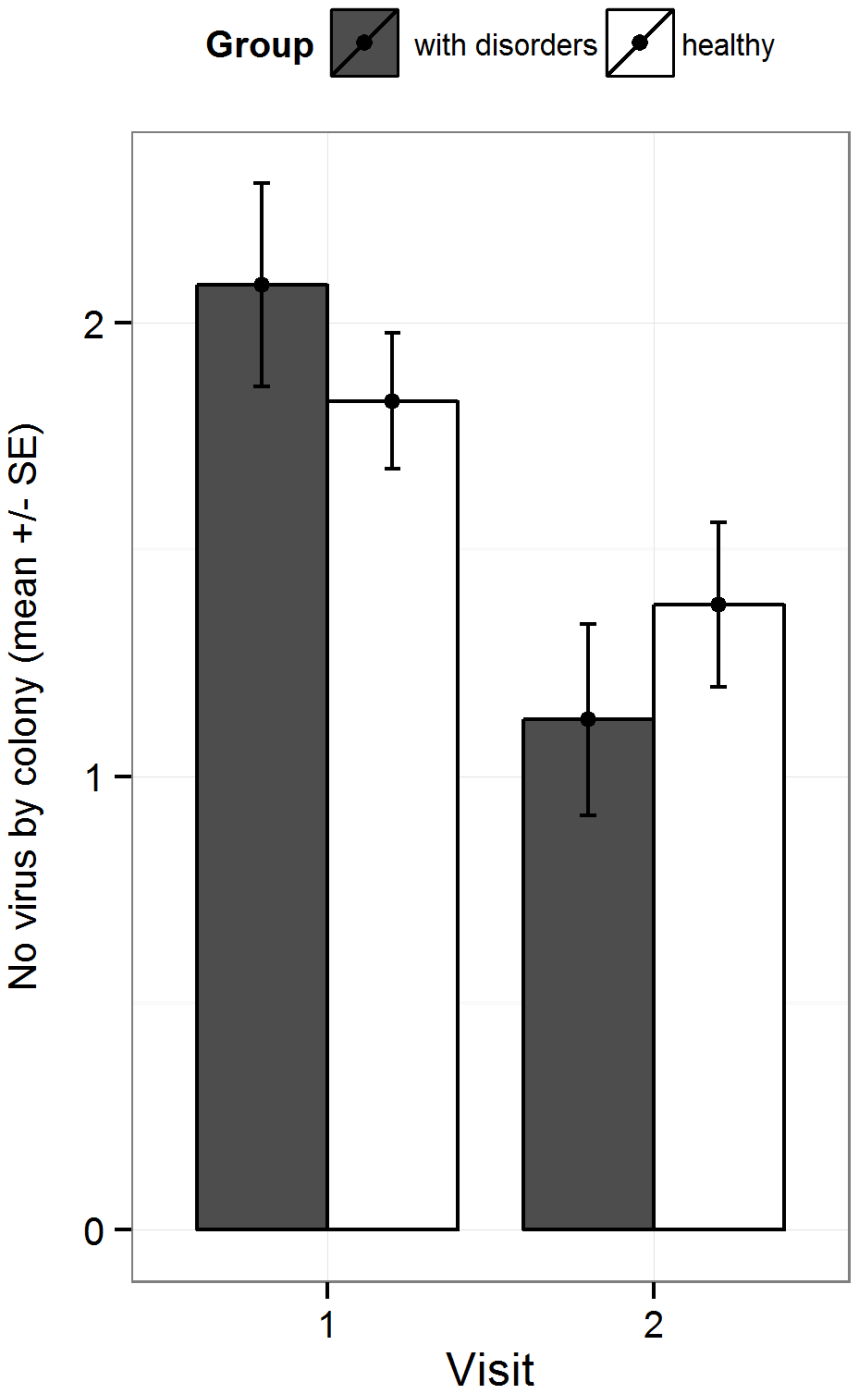
Average number of different viruses per colony. Data shown for the first and second visits (visit 1 - mid-July to mid-august and visit 2 - mid-September to mid-October) and the group with disorders (grey, n = 24 colonies) and the healthy one (white, n = 29 colonies). Whiskers show the standard error (SE).

There was no significant difference in viral content between the group with disorders or the healthy one, and independently of the visit for any of the three most abundant viruses (2-way ANOVA tested by permutations, DWV: p = 0.731, BQCV: p = 0.373, SBV: p = 0.54). We observed a decrease of virus content from summer to fall 2011, independently of the group, for BQCV (p<0.001) and SBV (p<0.001) but not for DWV (p = 0.271). For BQCV only, we observed a significant (p = 0.036) group×visit interaction indicating that the decrease of the virus abundance from visit 1 to visit 2 was higher in the group “with disorder” than in the group “healthy”. Overall, the number of virus changed between visit 1 and visit 2 and a significant decrease was observed in the group with disorders (p<0.001) (Figure 3).

### Pesticide analysis

**Figure 4:**
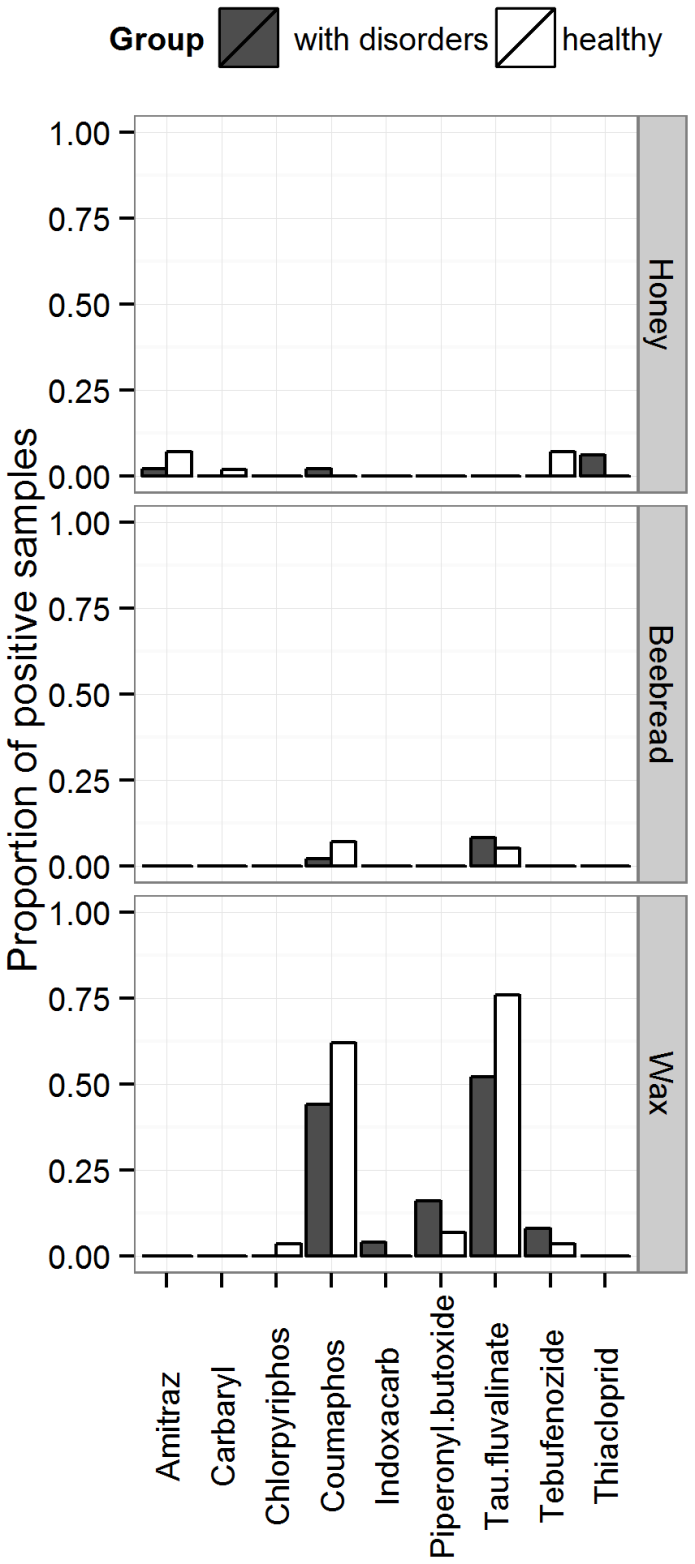
Proportion of samples containing residues of acaricides/insecticides in the different beekeeping matrices (honey, beebread and wax). Data shown for the group with disorders (grey, n = 25 colonies) and the healthy one (white, n = 29 colonies).

**Figure 5:**
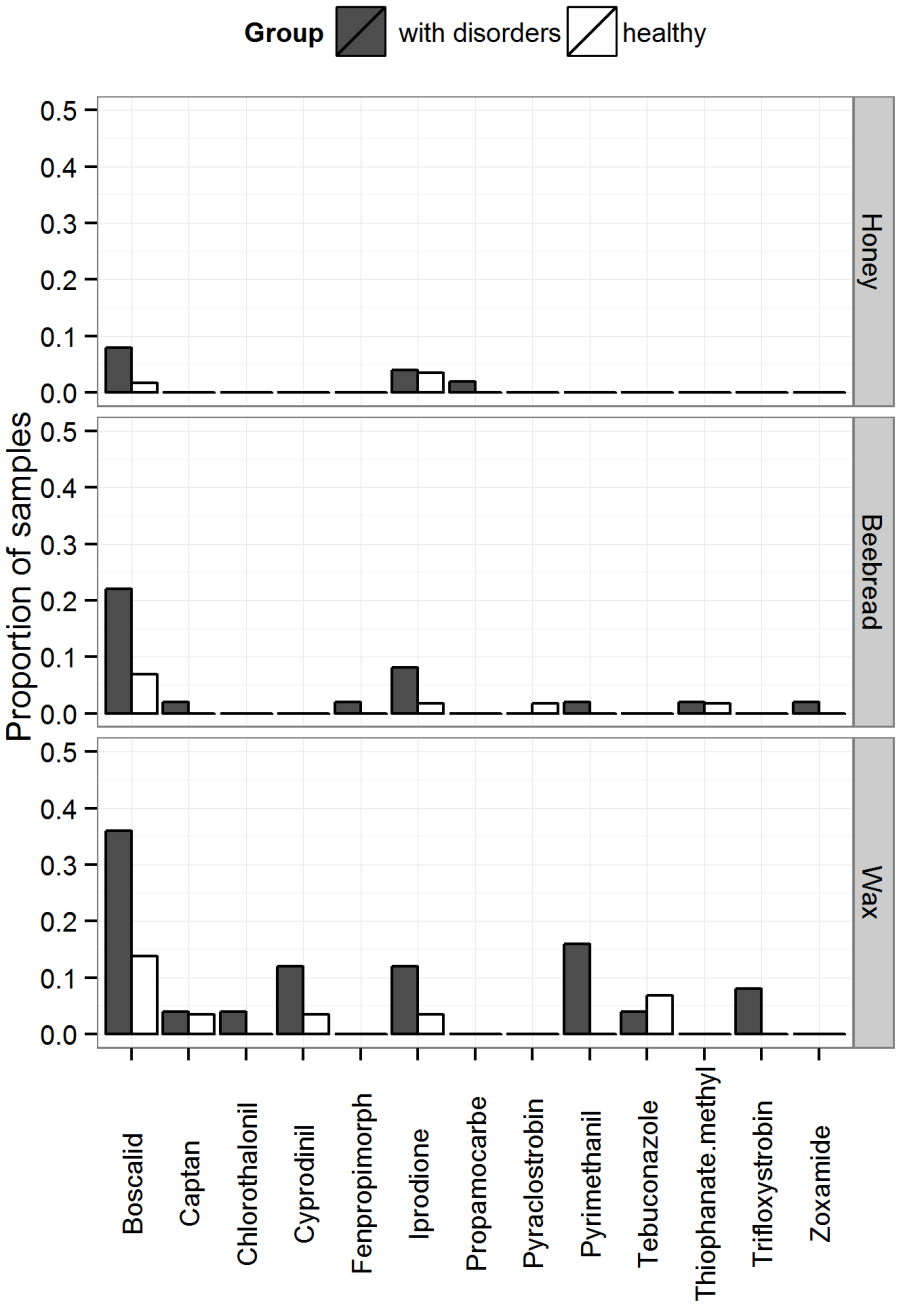
Proportion of samples containing residues of fungicides in the different beekeeping matrices (honey, beebread and wax). Data shown for the group with disorders (grey, n = 25 colonies) and the healthy one (white, n = 29 colonies).

172 agrochemical residues of 23 different active ingredients were detected in 94 out of 269 samples. Wax was the most contaminated matrix: 109 residues of 15 different active ingredients; while 39 and 24 residues of 10 and 8 substances were detected in beebread and honey, respectively (Figures 4 & 5). Residue levels contained in wax and beebread were higher (0.21–3.1 mg/kg) than those in honey (0.001–0.058 mg/kg).

For the subsequent statistical analysis, the results obtained for insecticide and acaricides residues were pooled together because the most frequently found active ingredient, tau-fluvalinate (n = 46), can be used in Belgium as an acaricide against varroa mite and as an insecticide to control *Meligethes aeneus* in rape. The second most frequently found residue was the coumaphos (n = 35), followed by two fungicides, boscalid (n = 33), iprodione (n = 13) (Table 4). Some residues of neonicotinoid insecticides, synergist and herbicide were also detected: thiacloprid (n = 3), piperonyl butoxide (n = 6), terbuthylazine (n = 1). The highest residue level concerned captan with 3.1 and 1.9 mg/kg in the wax and bee bread of the same colony, respectively. Despite of being the most frequently found in matrices, tau-fluvalinate and coumaphos residues never exceeded 0.71 and 0.58 mg/kg, respectively. Boscalid, the most commonly found fungicide, ranged from 0.005 to 1.3 mg/kg.

**Table 4:** Residues of active ingredients found in wax, beebread and honey samples from colonies with disorders (Group D, n = 25 colonies) and healthy ones (Group H, n = 29 colonies).

Activeingredient	Group	Wax							Bee bread							Honey						
		LOQ(mg/kg)	Number of samples (n = 54)			Residues amount (mg/kg)			LOQ(mg/kg)	Number of samples (n = 108)			Residues amount (mg/kg)			LOQ(mg/kg)	Number of samples (n = 107)			Residues amount (mg/kg)		
			<LOD	Detected	Quantified	Range	Mean	s.d.		<LOD	Detected	Quantified	Range	Mean	s.d.		<LOD	Detected	Quantified	Range	Mean	s.d.
Amitraz	D	0.1	–	–	–				0.1	–	–	–				0.01	49	–	1	0.022	0.02	
	H		–	–	–					–	–	–					53	–	4	0.01–0.028	0.02	0.01
Boscalid	D	0.1	16	8	1	0.29	0.29	–	0.1	39	7	4	0.4–1.3	0.68	0.42	0.005	46	–	4	0.005–0.026	0.01	0.01
	H		25	4	–	–	–	–		54	4	–	–	–	–		56	–	1	0.058	0.06	–
Captan	D	0.1	24	–	1	3.1	3.1	–	0.1	48	–	1	1.9	1.90	–	0.01	–	–	–			
	H		28	1	–	–	–	–		58	–	–	–	–	–		–	–	–			
Carbaryl	D	0.1	–	–	–				0.1	–	–	–				0.005	50	–				
	H		–	–	–					–	–	–					56	–	1	0.02	0.02	–
Chlorpyriphos	D	0.1	25	–	–				0.1	–	–	–				0.005	–	–	–			
	H		28	1	–					–	–	–					–	–	–			
Chlorothalonil	D	0.1	24	1	–				0.1	–	–	–				0.005	–	–	–			
	H		29	–	–					–	–	–					–	–	–			
Coumaphos	D	0.1	14	9	2	0.32–0.34	0.33	0.01	0.1	48	1	–				0.05	49	–	1	0.012	0.01	–
	H		11	10	8	0.23–0.58	0.37	0.01		54	4	–					57	–	–			
Cyprodinil	D	0.1	22	3	–				0.1	–	–	–				0.01	–	–	–			
	H		28	1	–					–	–	–					–	–	–			
Fenpropimorph	D	0.1	–	–	–				0.1	49	1	–				0.005	–	–	–			
	H		–	–	–					58	–	–					–	–	–			
Indoxacarb	D	0.1	24	1	–				0.1	–	–	–				0.005	–	–	–			
	H		29	–	–					–	–	–					–	–	–			
Iprodione	D	0.1	22	1	2	0.24–1.5	0.87	0.89	0.1	45	–	4	0.34–1.5	0.90	0.48	0.005	48	–	2	0.017–0.022	0.02	0.00
	H		28	1	–	–	–	–		57	1	–	–	–	–		55	–	2	0.022–0.04	0.03	0.01
Piperonyl butoxide	D	0.1	21	4	–				0.1	–	–	–				0.01	–	–	–			
	H		27	2	–					–	–	–					–	–	–			
Propamocarbe	D	0.1	–	–	–				0.1	–	–	–				0.005	49	–	1	0.008	0.01	0.00
	H		–	–	–					–	–	–					57	–	–	–	–	–
Pyraclostrobin	D	0.1	–	–	–				0.1	50	–	–				0.005	–	–	–			
	H		–	–	–					57	1	–					–	–	–			
Pyrimethanil	D	0.1	21	4	–				0.1	49	1	–				0.005	–	–	–			
	H		29	–	–					58	–	–					–	–	–			
Tau-fluvalinate	D	0.1	12	9	4	0.29–0.46	0.4	0.08	0.1	45	4	–				0.01	50	–	–	–	–	–
	H		7	13	9	0.21–0.71	0.5	0.08		55	3	–					53	–	4	0.011–0.02	0.02	0.00
Tebuconazole	D	0.1	24	1	–				0.1	–	–	–				0.005	–	–	–			
	H		27	2	–					–	–	–					–	–	–			
Tebufenozide	D	0.1	23	2	–				0.1	–	–	–				0.005	–	–	–			
	H		28	1	–					–	–	–					–	–	–			
Terbuthylazine	D	0.1	24	1	–				0.1	–	–	–				0.01	–	–	–			
	H		29	–	–					–	–	–					–	–	–			
Thiacloprid	D	0.1	–	–	–				0.1	–	–	–				0.005	47	–	3	0.009–0.013	0.01	0.00
	H		–	–	–					–	–	–					57	–	–	–	–	–
Thiophanate-methyl	D	0.1	–	–	–				0.1	49	1	–				0.005	–	–	–			
	H		–	–	–					57	–	1	0.38	0.38	–		–	–	–			
Trifloxystrobin	D	0.1	23	2	–				0.1	–	–	–	–	–	–	0.01	–	–	–			
	H		29	–	–					–	–	–					–	–	–			
Zoxamide	D	0.1	–	–	–				0.1	49	1	–				0.01	–	–	–			
	H		–	–	–					58	–	–					–	–	–			

**Figure 6:**
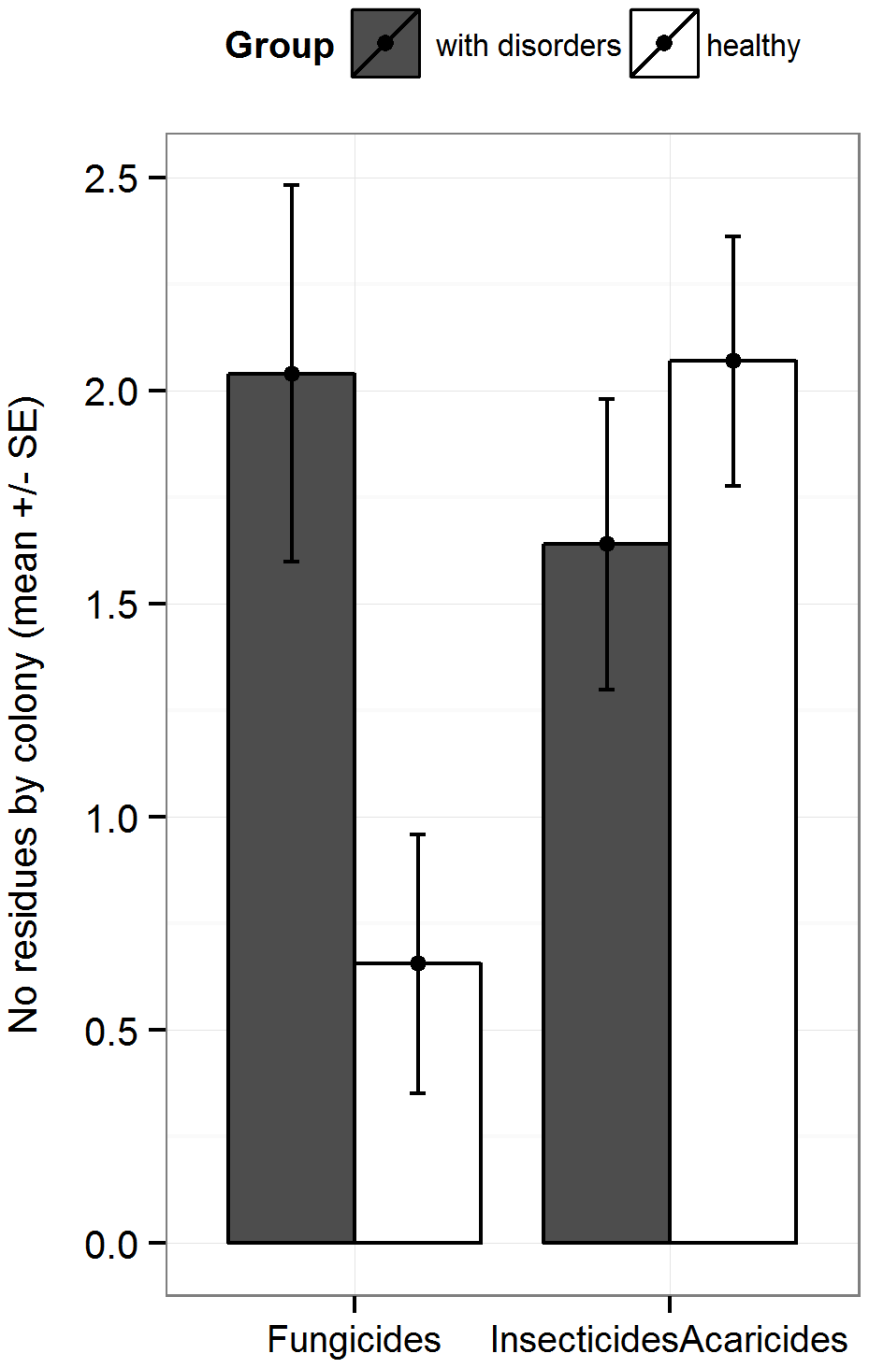
Average number of residues per colony. Data shown for the group with disorders (grey, n = 25 colonies) and the healthy ones (white, n = 29 colonies). Whiskers show the standard error (SE).

There was a significant difference (p = 0.01) in terms of number of fungicide substances found between the “disorder” group (mean = 2.0) and the “healthy” one (mean = 0.7) as presented in Figure 6. The total number of insecticides/acaricides residues was slightly higher in the disorder group (mean = 2.1) than in the healthy group (mean = 1.64) but this difference is not statistically significant (p = 0.79). The most frequent fungicides in the group with disorders were boscalid and iprodione, detected in the three investigated matrices (Figure 5). However, for single active substances, no significant difference of number of residues was observed between the groups “with disorder” and “healthy”.

### Probability of disorders in relation to potential stressors

**Figure 7:**
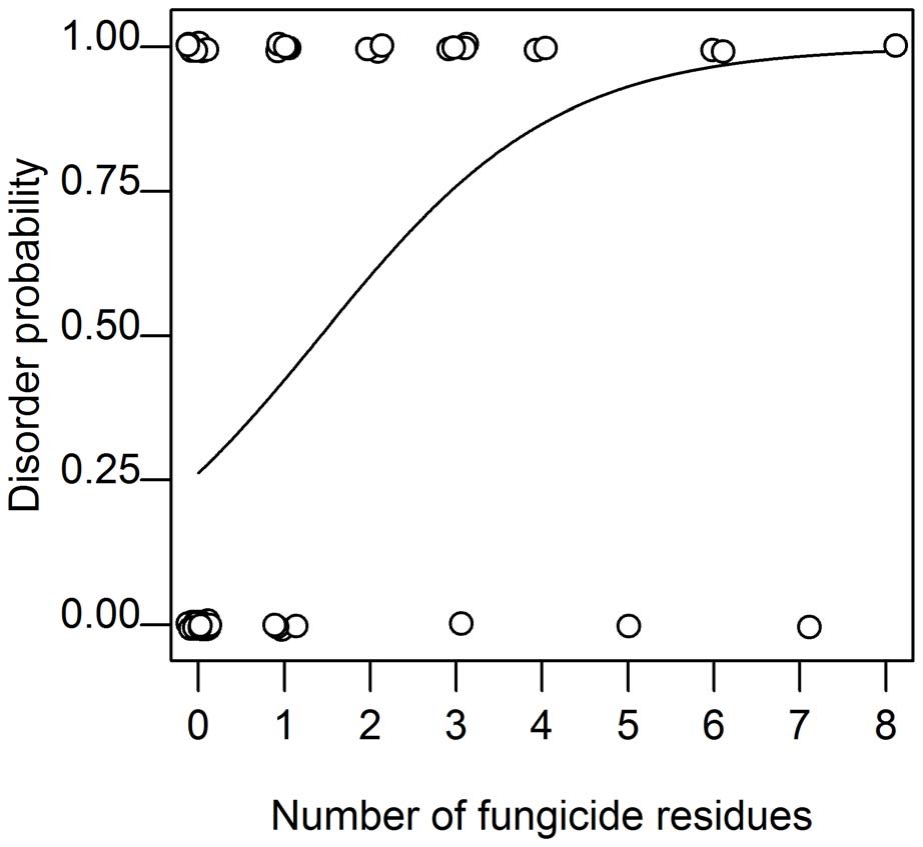
Probability of honeybee colony disorders depending on the number of fungicide residues detected. Model based on averaged coefficients and median value both for the number of insecticides-acaricides residues and total number of virus detected for both visits (n = 53 colonies).

There is clearly a significant positive relationship between the probability of a colony showing disorders and the total number of fungicides (Figure 7, LR = 7.128, df = 1, p = 0.008). The estimated probability for a colony to be in the “with disorder” group is 0.26 without fungicides residues, 0.60 with 2 fungicides residues and 0.88 with 4 fungicides residues when the insecticides-acaricides residues number and virus load are fixed to their observed mean value (Figure 7). None of the other variables (total number of viruses, total number of insecticides-acaricides) and none of the first level interactions seem to have any explanatory power on the probability of disorders in a colony (Table 5). No direct link could be established between bee colony disorders and the amount (in µg/kg) of residues found in the matrices.

**Table 5:** Analysis of deviance table for generalized linear binomial models describing the relationships between the colony disorder probability and three variables: the total number of (1) fungicide residues (totfungicides), (2) insecticide-acaricide residues (totinsaca) and (3) virus detected for both visits (totvirus).

	LR	df	p(>Chisq)
totfungicides	7.13	1	0.008
totinsaca	0.005	1	0.943
totvirus	0.136	1	0.712
totfungicides:totvirus	2.222	1	0.136
totinsaca:totvirus	0.975	1	0.323
totfungicides:totinsaca	0.901	1	0.342

LR = likelihood ratio.

### Probability of disorders and crop/grassland surface

**Figure 8:**
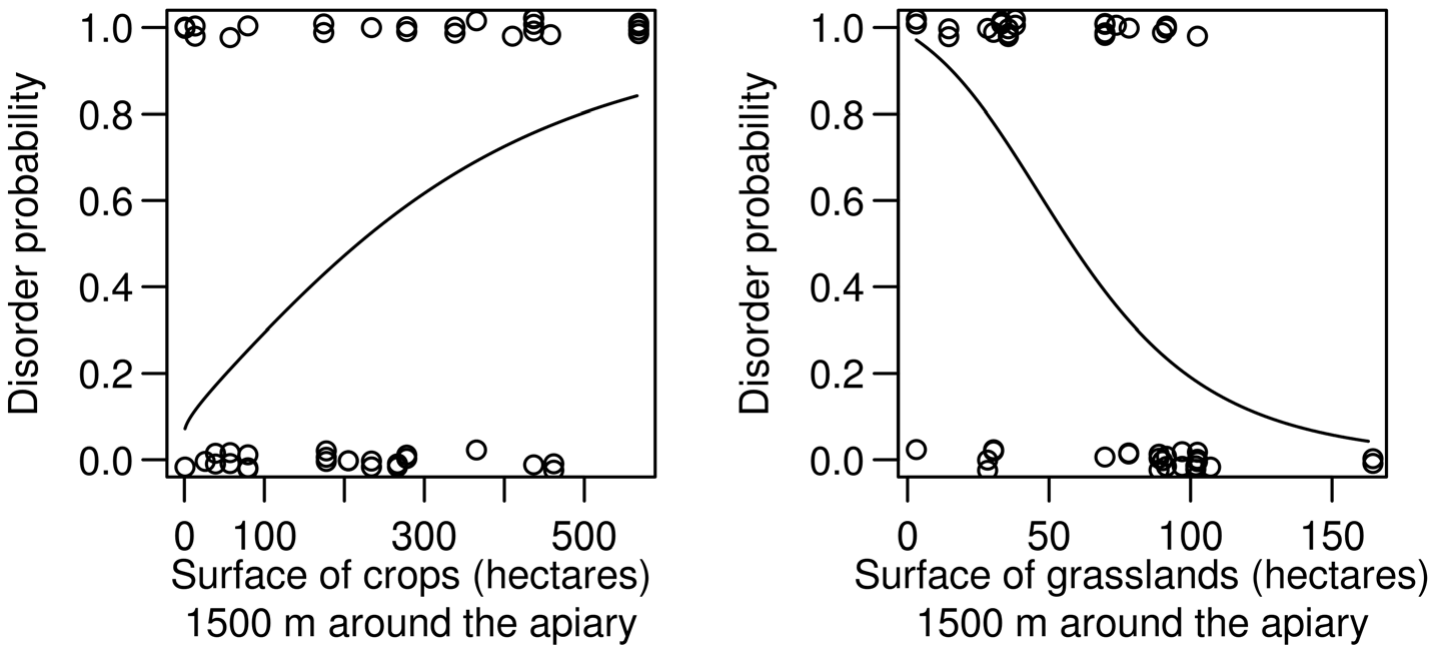
Probability of honeybee colony disorders depending on the apiary’s environment. Consideration of crop surface *vs.* grassland surface in a radius of 1500 m around the apiary. Crops include fruit, vegetables, fodder production and horticulture. For each graph, the value of the variable not displayed is fixed to its observed mean (n = 53 colonies).

Our data clearly show a significant increase in disorder probability with the increase of crop surfaces (*sensu lato*, i.e. including fruit, vegetables, fodder production and horticulture) in the surrounding of the apiary (Figure 8, LR = 8.052, df = 1, p = 0.0045). The predicted probability of disorders is close to 0.1 for a crop surface of 0 ha in the radius of 1500 m and increases up to 0.8 for a surface of 500 ha of crops when fixing the grasslands surface to its observed mean.

On the contrary, the probability of disorders strongly decreases when the grassland surfaces increases (Figure 8, LR = 14.527, df = 1, p = 0.0001) after controlling for the crop surface. The predicted probability of disorders is close to 1 for a grassland surface of 0 ha in a radius of 1500 m and drops to ∼0.1 for a surface of 150 ha of grasslands when fixing the crops surface to its observed mean.

Very similar results were found with a 3000 m buffer around the apiary (results not shown).

## Discussion

The bee disorders observed after the winter in the Walloon region happened despite of normal climatic conditions. The autumn of 2011 was dry (140.4 mm rainfall water from October to December, average being 219.9 mm), sunny and warm for Belgian conditions (12.4°C on average, which is normally 10.9°C for this period), followed by average winter and spring 2012 in terms of temperature, rainfall, with the exception of a cold wave lasting twelve days in February [53]. These conditions would not induce, *a priori,* a risk for honeybee colonies. Furthermore, based on studies carried out on the palynological diversity of the pollens collected by these colonies before the winter (Table S1), the hypothesis of nutritional lack is unlikely. All samples analysed contained at least 8 different botanic sources of pollen, including rich protein content as oilseed rape, ivy and *Phacelia*.

Viruses infections has often been mentioned as a source of stress for honeybee colonies [7]. However no significant difference in quantity or occurrence was observed between the healthy and the “with disorder” groups. DWV is one of the most commonly detected virus in *A. mellifera*. The prevalence of this virus is even more important at colonies infested by *Varroa destructor*, a well-established vector of this virus [7]. In accordance with the present study, further studies run on Belgian apiaries show DWV, BQCV and SBV as the most frequently found viruses [54]. Unlike DWV, we observed that the amount of SBV and BQCV has dropped significantly between the first and second visits for each group. In the case of BQCV, this is of no surprise as the cycle of incidence of this virus has been shown to increase in late winter, with a peak in May or June followed by a rapid decline [55]. The observed decrease of SBV has also been reported in other studies [42] in which the authors suggested that bees could develop a molecular defensive mechanism to reduce virus multiplication, or that the change in bee susceptibility to the virus could result from environmental conditions such as the quality of pollen.

We cannot prove any causal relationship between any of these viruses analysed and bee disorders, nor does the interaction between these two factors. Cox-Foster et al., 2007 [41] found no clear correlation between a variety of pathogens, including *Nosema* spp., DWV, CBPV, ABPV, BQCV, *Mellisococcus pluton* and *Paenibacillus larvae* ssp and CCD. No specific spore counts were carried out in our study. However, no signs of nosemosis or foulbrood could be linked to colonies presenting bee disorders. It is noteworthy that a monitoring run at Belgian level found *Nosema* spp spores in one out of four colonies [56]. Nevertheless, Cox-Foster et al., 2007 [41], show a positive correlation between IAPV and CCD, which *a priori* would not be relevant in our conditions given the low prevalence of IAPV in this country [27], [54]. A recent publication [36] shows a positive correlation between the presence of fungicides in pollen loads and the presence of spores of *Nosema ceranae*. We do not exclude the potential involvement of *Nosema* spp. in the case of bee disorders. However, in the framework of our study, *N. ceranae* seems either to play a role in the development of this weakening, while remaining asymptomatic, or not to play a decisive role in it.

When considering pesticide residues, no direct link could be established between bee colony disorders and the amount (in µg/kg) of residues found in the matrices. Neither could we identify any specific molecule as cause of bee disorders. Nevertheless, the study of the residue load of pesticides, specifically fungicides, opens new avenues for a better understanding of honeybee colony disorders.

Even if insecticides/acaricides were the most numerous residues detected in hives mainly in wax, no significant difference in the number of accumulated residues was observed between colonies with disorders and the healthy ones. Indeed the two most abundant active ingredients, tau-fluvalinate and coumaphos, came most probably from varroa control measures even if tau-fluvalinate is used as an insecticide (Mavrik 2F) against *Meligethes aeneus* in oilseed rape. These active ingredients seem to be a frequent outcome of residue analyses studies [57], [58], [39], [38], [40], [59]. Synergistic effects have been shown between acaricides and other molecules [33], [60]. Nevertheless, our modelling did not suggest any synergistic effects in the appearance of bee disorders occurring between residues of fungicides and insecticides-acaricides. Residues of synergist, piperonyl butoxide, were also detected that indicates a prior exposure to synthetic or natural pyrethroids even though residues were not found in the analysed matrix, probably due to a fast degradation of these active ingredients [61]. A non-authorised active ingredient was also detected: carbaryl, forbidden in Belgium since 2007, indicating an illegal agricultural or gardening use.

The only neonicotinoid found, thiacloprid, was detected in honey during the sampling period of July-August in two colonies. However, the limits of detection achieved in our study do not allow to determine the presence of neonicotinoids -with the exception of acetamiprid- or fipronil at levels in the range of the acute toxicity of these substances (30–40 µg/Kg). Residues of these substances could be present at lower levels and thus an exposure to these substances cannot be excluded.

According to our results, the number of fungicide residues seems to plays a role in the appearance of honeybee colony disorder.

Significantly, more fungicides residues were detected in colonies with disorders than in the healthy ones. Mainly four active ingredients, frequently used in plant protection, were detected: boscalid, iprodione, pyrimethanil and cyprodinil. Fungicides are often considered safe for honeybees based on their acute toxicity. However some direct toxic effects on bees either by the mother compounds or their metabolites have been reported in the past [62]. For example, boscalid shows low acute toxicity to bees [63], despite the fact that beekeepers in the USA have already reported losses and adverse effects on bee brood development related to the foliar application of this systemic active ingredient [64]. Incidents were often linked to the use of a co-formulation boscalid – pyraclostrobin. However, both molecules appeared above detection levels in only two colonies of our study and could not explain the general trend. Interestingly, one metabolite of boscalid, the 2-chloronicotinic acid, is similar to the 6-chloronicotinic acid, a metabolite of imidacloprid. The latter has proved to be lethal to bees at low concentrations (0.1 µg/L) following chronic exposure [65]. Further research should be carried out in order to clarify the mechanism of bee toxicity of boscalid. Indeed, boscalid has proved toxic to other aquatic invertebrates, reducing daphnid fecundity and *Chironomid* emergence [64]. In addition, synergism with other active ingredients like insecticides are possible and increase the toxicity for honeybee [66], [32], [67]. Two other fungicides were also detected at very high levels in wax and beebread: iprodione and captan. The former is known for its synergistic effects in collaboration with insecticidal compounds [33]. The latter has been shown to induce effects on growth and development of larval honeybees [68], [69]. Chronic and larval toxicity studies would be interesting at this stage in order to evaluate possible direct toxic effects on bee individuals and their behaviour. Indeed, a recent study showed increased larval mortality following chronic exposure to tau-fluvalinate, coumaphos, chlorpyriphos and chlorotalonyl or some of their combinations [70]. All these substances were found in our study. Increased mortality rates in the fall may compromise the size and age structure of the wintering cluster, which could lead to winter losses.

The indirect effects of fungicides on bees or on bee colonies are relatively little-known to date.

Fungicides may have an impact on the colony by modifying the existing microflora present in the food stores or in the bee intestinal tract [71]. Studies have already shown the possible modification of microbial composition both at beebread level [62], [72] and at intestinal level [73]. This modification in the composition of microbiota may lead to dysbiosis [74]. The impact that such an unbalance in the bee gut microflora may have on bee health has already been considered. The link between the unspecific symptoms observed in our study and a possible microbial alteration could be subject of further research.

In parallel, the potential impact of microbial modification on digestibility and availability of nutrients should be a target for further research. Indeed, the content of essential amino acids might be altered when beebread is contaminated with fungicides (DeGrandi-Hoffman, 2013, pers com.). Given the importance of nutrition, especially pollen, in the good development of the colony [75] alterations in composition or lack of essential nutrients would put the homeostasis of the colony at stake. Some studies have already shown the impact of nutritional lack on bee development and health [76]. Provided that pollen is the unique source of amino acids for honeybees, royal jelly production could also be affected [77], [78] with unexpected potential consequences for its main consumers, larvae and the queen. A poor nutrition of the queen, could have as a result an impact in its activity. Likewise, a poor nutrition of the larvae has been shown to impact their development [76]. As a result, the presence of fungicides on beebread and honey may have both a direct effect on their health, but also an indirect one on the colony development.

Fungicides are widely used in agriculture and are broadly present in bee matrices, sometimes at high concentrations at levels of mg/kg [79], [80], [38], [59]. Boscalid, cyprodinil, iprodione are used to control a broad range of fungal pathogens including *Botrytis* spp., *Alternaria* spp. and *Sclerotinia* spp. on a wide range of crops including fruits, vegetables and ornamentals. Pyrimethanil is more specifically used to control grey mould on fruits, vegetables and ornamentals, and leaf scab on apple trees [81]. These active ingredients were already reported in bee matrices in Europe and in the USA [23], [39], [62]. Their frequent presence in bee matrices might be an indication of chemical plant protection intensity in all agricultural landscapes. Fungicides could also be markers of exposure to other active ingredients with higher toxicity to bees. Mixes of products like fungicides – insecticides are often applied to reduce the number of spray applications. As a result, other pesticides often used in combination with the fungicides found or applied in the same crops could have been at the origin of the effects observed in this study. However, the sensibility of the residue analyses used in our study might explain the lack of detection of such components. Further intensive monitoring and a thorough record of the agricultural practices on pesticide application in tank mixes would help clarifying this alternative explanation.

Factors different from fungicides are most likely involved in the development of bee disorders. According to our model, the disorder probability was not absent when fungicides residues were not detected in presence of insecticide residues and virus. Non identified pathogens, chemicals or factors of different nature could be operating as silent stressors. Ravoet and colleagues (2013) [82] reported the presence of new pathogens in Belgium that were not taken into consideration in our analyses (i.e. *Crithidia mellificae* or the Lake Sinai Virus (LSV)). Other stressing factors could be also linked to the intensive agriculture. In fact, we observed an increase of colony disorders in the area with high density of crops in comparison with areas with grassland. Some studies and beekeeper claims go in line with our outcome regarding the concentration of bee problems in areas with intensive agriculture [83]–[86]. Furthermore, the same negative trends on pollinators and biodiversity in areas with intensive agriculture have already been described as the result of as increased pesticide use, decreased landscape heterogeneity, loss and fragmentation of natural habitat [87], [88].

In conclusion, the five virus studied (ABPV, CBPV, QBCV, SBV, DWV) do not seem determinant in the appearance of bee disorders in our study. These disorders seem clearly linked to the environment of the apiaries and were observed mainly in agricultural crop areas. We observed also that the number of fungicide residues appears as the main potential stress factor linked to bee disorder. However other stressing factors could be acting or interacting at the same time: insecticides exposure, a lack of amino acids and oligo-elements, etc. Our results open new avenues for future research in order to better understand the side effects of fungicides on the bee colony and questions the sustainability of intensive agriculture model and its impact on bees. Specific toxicological studies on both adult bees and larvae would be recommendable in order to better characterise the toxicity of fungicides.

## Supporting Information

Table S1 **Diversity of pollens collected in the apiaries before the winter.**
(DOC)
