# Supplementary material for: Honeybee Colony Disorder in Crop Areas: The Role of Pesticides and Viruses
Source: PLoS One. 2014 Jul 21;9(7):e103073. doi: 10.1371/journal.pone.0103073 (PMC4105542; doi:10.1371/journal.pone.0103073)
Supplement: Table S1 — Diversity of pollens collected in the apiaries before the winter. (DOC) [file pone.0103073.s001.doc]

**Table S1. Diversity of pollens collected in the apiaries before the winter.**
